# A seasonally explicit counterfactual framework reveals incomplete turbidity recovery in a tropical river seven years after a major mine-tailings disaster

**DOI:** 10.1007/s10661-026-15706-2

**Published:** 2026-08-20

**Authors:** Juliana S. Leal, Natália F. Souza

**Affiliations:** 1 Programa de Pós-Graduação Em Ecologia E Evolução, Universidade Do Estado Do Rio de Janeiro (UERJ), Rio de Janeiro, RJ Brazil; 2 Departamento de Ecologia, Instituto de Biologia, Universidade Federal Do Rio de Janeiro (UFRJ), Cidade Universitária, Rio de Janeiro, RJ Brazil; 3 Associação Pró-Gestão das Águas da Bacia Hidrográfica Do Rio Paraíba Do Sul (AGEVAP), Secretaria Executiva Do Comitê Piabanha, Petrópolis, RJ Brazil; 4 Programa de Pós-Graduação Em Ecologia, Universidade Federal Do Rio de Janeiro (UFRJ), Rio de Janeiro, RJ, Brazil

**Keywords:** Doce River, Fundão Dam, Suspended sediments, Lotic ecosystems, Water quality, Environmental impact assessment, Quasi-experimental design

## Abstract

River channels have been extensively impacted by tailing inflows worldwide. Evaluating the environmental impacts of tailing dam failures using methodologically rigorous, time-series–based approaches is an essential step in supporting recommendations for the design of long-term monitoring and management actions. In southeastern Brazil, the 2015 Fundão tailings dam failure discharged approximately 43 million m^3^ of iron ore tailings into the Doce River. Several studies have shown that turbidity is the most conservative metric for assessing water quality recovery in the Doce River. Here, we analyzed a 26-year turbidity record from 13 monitoring stations along the upper and middle reaches of the Doce River to quantify and characterize the response to the Fundão tailings dam failure seven years after the event. To do this, we used an integrated framework for trend analysis of environmental monitoring data, combining seasonal decomposition, interrupted time-series models with counterfactual “no-impact” scenarios, upstream–downstream contrasts, and standardized effect sizes. From a more conservative perspective, we showed that turbidity recovery was not complete by 2022; it remained partial, largely confined to the dry season and the furthest downstream portion of the evaluated reach of the Doce River. We argue that post-failure evaluation in the Doce River, and likely in other tailings-impacted rivers, should maintain high-frequency, long-term measurements, with particular emphasis on the rainy months and stations closest to the mine tailings spill site.

**Supplementary Information:**

The online version contains supplementary material available at https://doi.org/10.1007/s10661-026-15706-2.

## Introduction

Mines generate billions of tons of tailings annually, which are commonly stored as slurries in surface reservoirs (Cotrina-Teatino et al., 2025). When abruptly discharged, these tailings form gravity currents, and freshwater ecosystems provide preferred corridors for their spread (Hamilton et al., 2020). Globally, at least 5,300 km of river channels have been impacted by tailing inflows (Macklin et al., 2023). Tailings vary with the ore type and processing route but are typically fine-grained because of milling, which helps maintain them in suspension. Accordingly, affected freshwaters often show elevated turbidity (Abbadi & Mucsi, 2024; Opitz et al., 2020; Wolkersdorfer & Mugova, 2022). Turbidity is a key, low-cost water quality indicator used to diagnose particle interference with light transmission, evaluate water treatment performance, and is recognized for strongly influencing freshwater ecosystem functioning (Matos et al., 2024).

In freshwater ecosystems, turbidity often exhibits a natural seasonal cycle, changing dramatically between dry and rainy periods, typically peaking in the wet season as higher discharge and runoff mobilize and resuspend fine sediments. Nevertheless, tailing inputs can amplify these natural dynamics, producing turbidity peaks that far exceed seasonal background levels. Sustained time-series monitoring of turbidity in tailings-affected freshwater ecosystems is essential for disentangling natural seasonal variability from tailings signals, quantifying the magnitude and frequency of exceedances, and anticipating adverse effects on aquatic communities. However, the temporal scale is important for time-series monitoring of turbidity in tailings-affected freshwaters.

Over multi-year horizons, physical, chemical, and biological processes can drive the natural attenuation of turbidity (Younger & Wolkersdorfer, 2004). However, attenuation is not spatially uniform along the river network: as tailings are advected and deposited downstream, reaches that were initially clearer may later experience increased turbidity (Lauer & Parker, 2008). A robust approach for monitoring turbidity evaluates, beyond seasonal baselines, whether the downstream turbidity trends align with those upstream, indicating an actual recovery rather than the displacement of impacts.

Baseline-poor, control-free snapshot approaches to monitoring water quality parameters hamper inferences about the magnitude, duration, and spatial footprint of departures from background conditions (Hamilton et al., 2020; Helsel et al., 2020). Best practice in the time-series analysis of water quality parameters is to model step (level) changes in the series mean while accounting for autocorrelation and seasonality (Helsel et al., 2020; Hyndman and Athanasopoulos, 2021; Stone et al., 2024). However, these practices have been inconsistently implemented across tailings-spill studies and regional water quality reports (Crioni et al., 2023; Diem & Orimolade, 2024; Macklin et al., 2023; Schreiber et al., 2022). Simultaneously, there is an increasing recognition of the importance of Interrupted Time Series (ITS) for assessing environmental impacts (Wauchope et al., 2021).

The ITS framework is recommended in ecology as a robust quasi-experimental approach for evaluating environmental impacts when randomized experiments are infeasible (Wauchope et al., 2021). An ITS framework uses models that simultaneously represent pre- and post-intervention trends and any abrupt shift at the intervention, and it requires sequential observations with a recognizable temporal pattern and a well-defined disturbance date (Gianicolo et al., 2021; Wauchope et al., 2021). In addition, recent work on environmental data time series has emphasized that, besides the importance of explicitly separating long-term trends from seasonal structures, it is crucial to compare observed trajectories with counterfactual “no-impact” scenarios when evaluating disturbance effects (Grace et al., 2021; O’Garra et al., 2025; Schäfer et al., 2021). However, to date, this approach has not been used to assess the impacts of one of the largest recent tailings-dam collapses worldwide on freshwater ecosystems.

In 2015, the Fundão tailings dam collapse, widely regarded as one of the most severe environmental disasters in global mining, had profound impacts on the Doce River (Southeastern Brazil; Carmo et al., 2017). Approximately 43 million m^3^ of iron ore tailings were discharged into the Doce River, leading to extensive downstream deposition and marked increases in suspended sediment and metal loads across the water column and sediment (Carmo et al., 2017; Hatje et al., 2017). Tailings caused an abrupt deterioration in water quality in the Doce River, but studies suggest a subsequent recovery, with turbidity being the most conservative metric for assessing it (Garcia et al., 2024; Silva et al., 2022; Wild et al., 2024). Several studies have used long-term monitoring data to assess the impact of the Fundão tailings dam failure on water quality and to infer recovery trajectories (e.g., da Cunha Richard et al., 2020; Garcia et al., 2024; Silva et al., 2022). However, to our knowledge, no published study has decomposed long-term turbidity records into trend and seasonal components and then used seasonally explicit interrupted time-series models with upstream–downstream contrasts and standardized effect sizes to quantify the magnitude and persistence of departures from pre-failure baselines along the mainstem of the Doce River.

In this study, we analyzed a 26-year turbidity record from 13 monitoring stations along the upper and middle Doce River to quantify and characterize the response to the Fundão tailings-dam failure, considering the pre- and post-failure periods. Specifically, we aimed to (i) assess how the dam failure affected the components of the turbidity time series, namely the long-term trend and the seasonal structure; (ii) quantify the magnitude and persistence of the tailings dam failure’s impact on turbidity by comparing observed post-event values with counterfactual trajectories from a seasonally explicit interrupted time-series model and their associated effect sizes; and (iii) evaluate how the strength of this impact varies along the river continuum. To address these objectives, we compiled and quality-controlled long-term agency records, established site-specific seasonal baselines, and applied a consistent modeling framework across stations with upstream–downstream comparisons to detect departures and their trajectories. We then synthesized the results to describe the downstream patterns of impact magnitude and spatial footprint.

## Methods

### Study site and database

The Doce River drains an approximately 86,715 km^2^ catchment spanning the states of Minas Gerais (upper/middle reaches) and Espírito Santo (lower reaches). Its basin has been historically impacted by anthropogenic pollution (i.e., domestic and industrial sewage) (Macêdo et al., 2024) and pronounced land use changes (Rudorff et al., 2018). Its native vegetation stems mainly from the Atlantic Forest, with smaller remnants of the Cerrado, both of which are biomes that are heavily threatened by agricultural expansion (Carvalho Ribeiro et al., 2020). Areas formerly covered by native vegetation have been widely converted to urban, agricultural, or mining land uses (ANA, 2016). Unregulated deforestation and poor soil management have accelerated erosion across the region, resulting in high suspended sediment loads and elevated turbidity in the Doce River, particularly during the rainy season (ECOPLAN-LUME, 2010). Approximately 86% of the basin area lies within the state of Minas Gerais (Aires et al., 2022), which operates a statewide water quality monitoring program through the Minas Gerais Water Management Institute (IGAM).

**Fig. 1:**
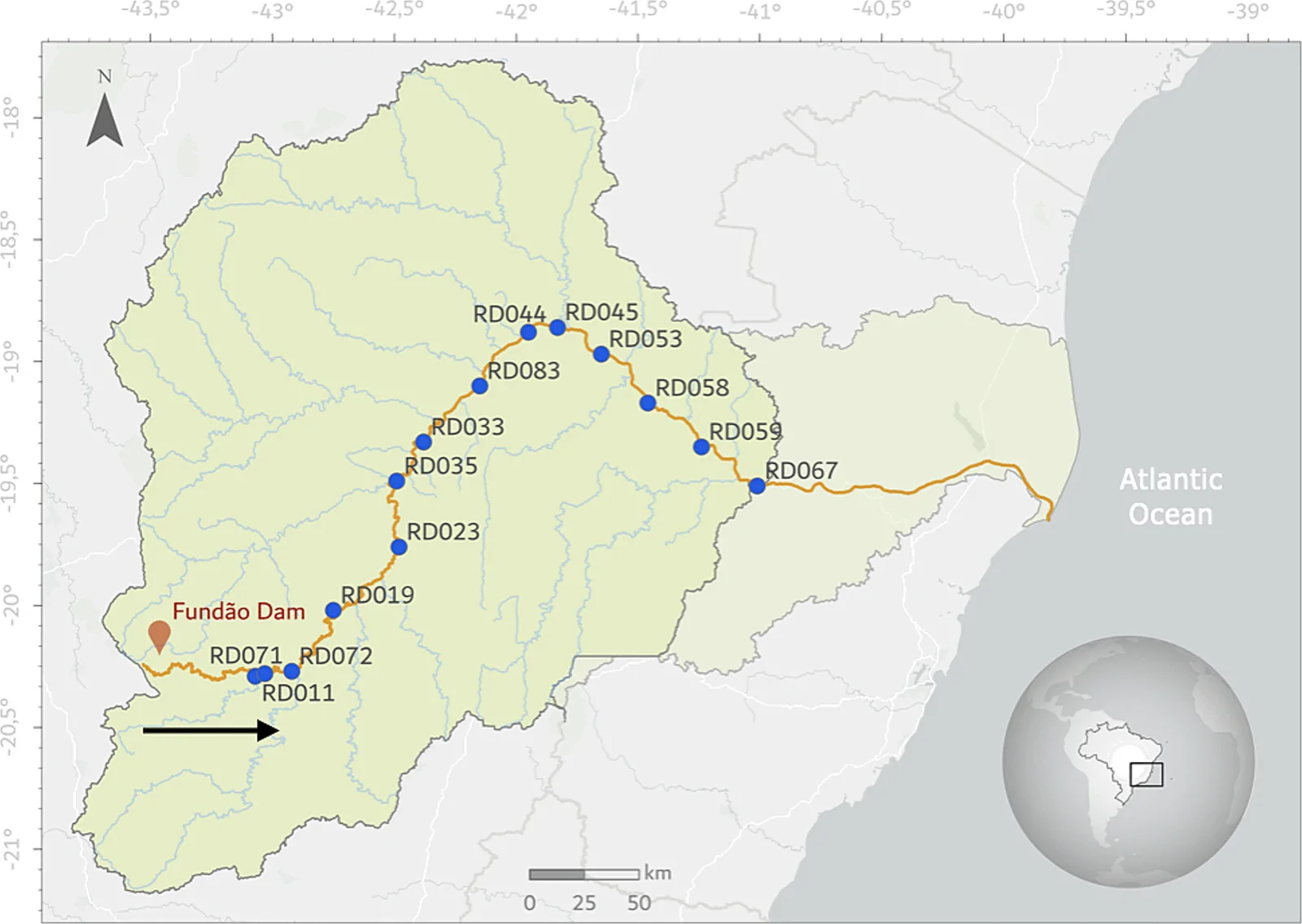
The Minas Gerais Water Management Institute (IGAM) monitoring stations, located along the section affected by the dam failure, were selected for our turbidity time-series analysis. The black arrow indicates the Doce River flow direction

Through its hydrometric and water quality network, IGAM regularly monitors physicochemical parameters (e.g., turbidity), building long time series that support basin-scale assessments. From 65 georeferenced IGAM monitoring stations in the Doce River Basin, we selected 13 (Fig. 1) for our time-series analysis because they were positioned downstream of the Fundão failure and had available pre-failure turbidity records. Turbidity data were obtained from the IGAM InfoHidro platform (http://portalinfohidro.igam.mg.gov.br). The 13 monitoring stations retained in the analysis spanned 490 km of the Doce River—more than 70% of the impacted section— and provided a ~ 26-year time series (1997–2022), except for stations RD071, RD072, and RD083. These three monitoring stations provided shorter pre-failure time series (ranging from 6 to 10 years), but we included them in the analysis to gather sufficient data for time-series description and modeling purposes.

### Data analysis

To highlight the impact of the Fundão tailings dam failure on the turbidity of the Doce River, we used turbidity records from 1997 to 2022 at each monitoring station. The turbidity time series was aggregated to a monthly frequency and log-transformed to stabilize variance and improve residual normality. Missing observations were imputed using seasonally adjusted linear interpolation (Hyndman and Athanasopoulos, 2021). To do this, we first computed a Seasonal-Trend decomposition procedure based on Loess (STL; Cleveland et al., 1990) to estimate the annual seasonal component and then linearly interpolated the seasonally adjusted series. Stationarity was evaluated using the Dickey–Fuller test (Dickey & Fuller, 1979, 1981; Hamilton, 1994). Residual diagnostics included graphical inspection to assess normality and autocorrelation and partial autocorrelation function plots (ACF/PACF), along with Ljung–Box tests, to evaluate autocorrelation (Burns, 2022).

Given the expected seasonal variability in turbidity (Santana et al., 2021), the data were grouped into dry (April–September) and rainy (October–March) seasons to compare annual means and maxima over time. The time-series trend and seasonality were assessed using the Seasonal-Trend decomposition procedure based on Loess (STL; Cleveland et al., 1990) because turbidity data are expected to show recurrent seasonal variation as well as non-linear long-term changes associated with hydrological variability and disturbance effects. The trend component was used to identify long-term increases or decreases, and the seasonal component assessed recurrent annual patterns. We performed STL decomposition separately for both pre-failure and post-failure data to estimate seasonal and trend components independently for each period.

To quantify the impact of the Fundão tailings-dam failure on the turbidity of the Doce River, we applied an Interrupted Time Series (ITS) analysis. ITS is a regression-based time-series model designed to assess the effect of an intervention or event occurring at a clearly defined point in time (Schaffer et al., 2021; Turner et al., 2021a, 2021b). The failure impact was estimated by comparing the post-failure turbidity values with the counterfactual values generated by the model, which represent the predicted turbidity trajectories under the assumption that the event had not occurred, thus preserving the pre-failure trend. The ITS linear regression model was applied using the Ordinary Least Squares (OLS) method (Travis-Lumer et al., 2022; Turner et al., 2021a, 2021b). It included one variable representing the level change and another representing the slope change after failure, which can be formalized as follows:

$$Y_t=\beta_0+\beta_1\cdot time+\beta_2\cdot intervention+\beta_3\cdot time\;since\;intervention$$

where β_0_ is the pre-failure baseline level, β_1_ the temporal trend coefficient, β_2_ the post-failure level shift, and β_3_ the post-failure slope change. The variable *time* represents the elapsed time since the beginning of the series; *intervention* indicates whether the period is before (0) or after (1) tailings dam failure; and *time since intervention* captures the interaction between *time* and *intervention*, measuring how temporal change differs before and after the event. In addition to *time* and *intervention*, we included a Fourier Seasonal Term, pairs of sine and cosine functions at different frequencies, to account for seasonal patterns in the series (Miratrix, 2022). For counterfactual-based models, we assumed an ITS model with no slope change (i.e., *β*₃ = 0) or no level change (i.e., *β*₂ = 0), depending on the intervention specification (Helfenstein, 1991; Lopez Bernal et al., 2016). In modeling, the intervention model for the Fundão dam failure encoded a priori assumption about how this event affected turbidity: it allowed both an immediate level shift in turbidity following the impact and a change in slope, with turbidity decreasing in the years thereafter.

The effect size was computed as Cohen’s *d*, which is the ratio of the overall mean difference in the post-failure period to the pooled pre- and post-failure standard deviation (Cohen, 1988; Travis-Lumer et al., 2022). For each time point in the post-failure period, the mean difference was calculated by subtracting the model prediction under no impact (counterfactual values) from the model-based values. We then averaged these differences to obtain the overall mean difference for the entire intervention period. Finally, we divided this overall mean difference by the pooled standard deviation of the predictions (Travis-Lumer et al., 2022). Cohen’s *d* indicates how many standard deviations separate the two groups—in this case, the pre- and post-failure periods. Larger Cohen’s *d* values reflect stronger effects, with negligible effects when *d* < 0.2, small when *d* = 0.2–0.3, moderate when *d* = 0.5–0.8, and large when *d* > 0.8 (Cohen, 1988, 1992; Hedges & Olkin, 1985; Navarro, 2015).

## Results

We observed an abrupt increase in turbidity at all monitoring stations following the Fundão tailings-dam failure in November 2015 (Supplementary Material, Figure S1). Across monitoring stations, the post-failure period featured elevated turbidity in the first years, especially in 2015–2016, followed by a gradual decline after 2017 (Supplementary Material, Figure S1). Following the Fundão tailings-dam failure, the rainy season (October–March) exhibited excessive turbidity (> 400 NTU), with values reaching up to ten times higher than those observed before the failure (Supplementary Material, Figure S2). In contrast, during the dry season (April–September), the maximum turbidity remained similar to the pre-failure conditions (< 100 NTU) (Supplementary Material, Figure S2).

**Fig. 2:**
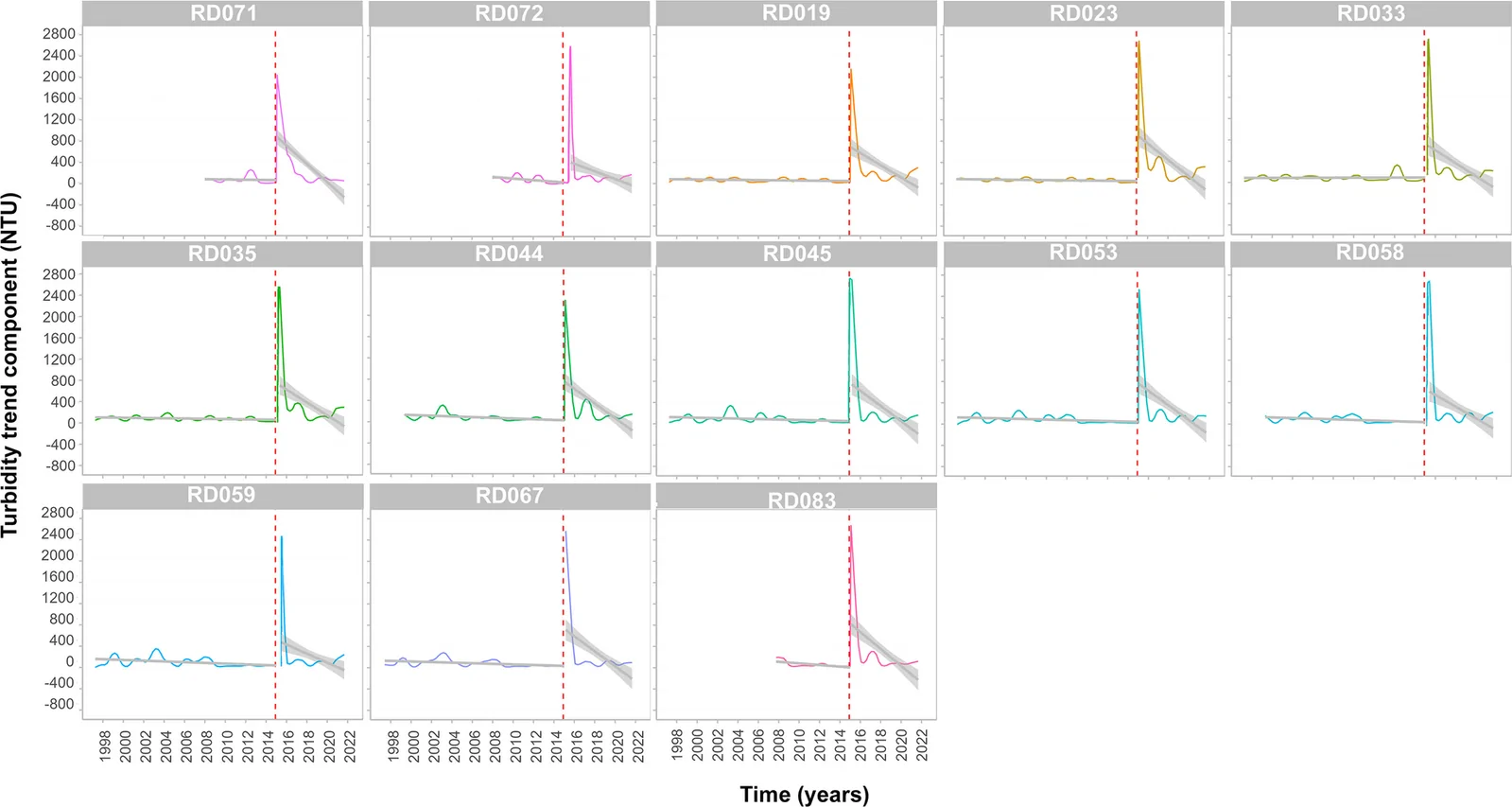
Trend component from the seasonal trend loess (STL) decomposition of monthly turbidity for each monitoring station along the Doce River reach impacted by the Fundão Dam failure. coloured lines show the STL trend component at each station, and grey lines with shaded bands indicate the fitted trend and its uncertainty. the red dashed vertical line marks the year of the Fundão dam failure (2015)

We assessed the turbidity time-series behavior through its trend and seasonality using Seasonal Trend Loess (STL). For the turbidity time-series trend, the STL showed a slight downward or essentially flat trend during the pre-failure period at all monitoring stations, but it abruptly increased and shifted to a marked downward trend in the post-failure period (Fig. 2). Despite this downward trend, we observed a much wider confidence interval for the post-failure period, indicating greater turbidity variation than that in the pre-failure period (Fig. 2).

**Fig. 3:**
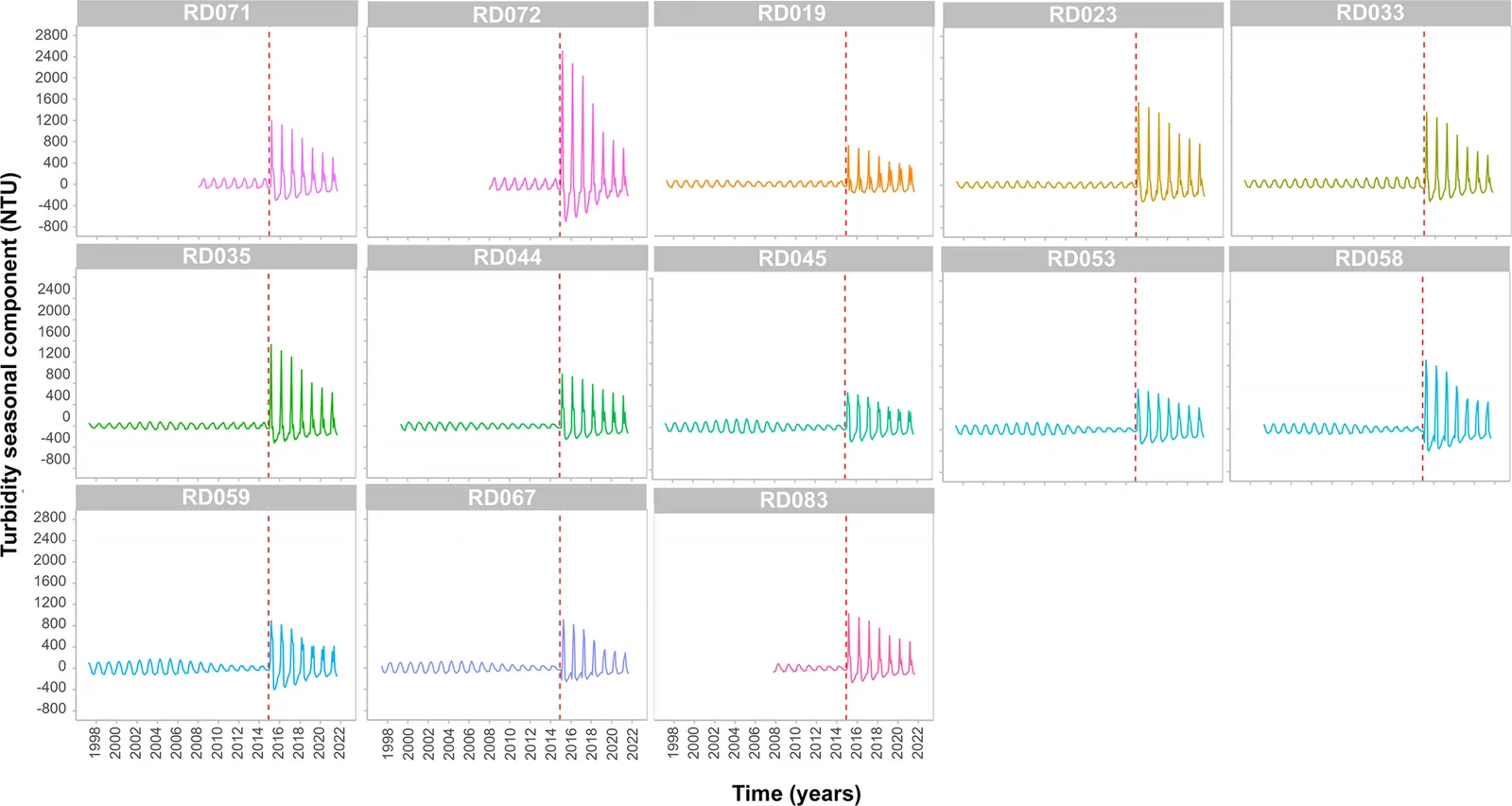
Seasonal component of the seasonal trend loess (STL) decomposition of turbidity for each monitoring station along the Doce River. within each panel, the recurrent peaks (rainy months) and troughs (dry months) represent the intra-annual wet–dry turbidity contrasts; their post-failure amplification indicates stronger seasonality after the Fundão Dam collapse. the red dashed line marks the year of the collapse (2015)

Regarding seasonality, we observed that it strongly influenced turbidity at all monitoring stations in both the pre- and post-failure periods (Fig. 3), with higher turbidity in the rainy months (October–March) and lower values in the dry months (April–September). The STL showed a greater seasonal amplitude in turbidity during the post-failure period, with sharper dry–wet contrasts in the years immediately following collapse, followed by a gradual attenuation over time (Fig. 3). However, this attenuation was insufficient to equalize the post-failure turbidity seasonal amplitude with that of the pre-failure period at any monitoring station. All fitted models satisfied the stationarity and residual assumptions (Supplementary Material, Table S2), indicating that the STL specification was adequate.

The Interrupted Time Series (ITS) model was statistically significant for all monitoring stations, and its R^2^ ranged from 0.45 to 0.60 (p < 0.001) (Supplementary Material; Table S1). The intervention coefficient indicated a significant post-failure level shift in turbidity at all monitoring stations, with increases of 150%–1800% relative to pre-failure values; that is, mean turbidity was approximately 2.5–19 times higher after the event (Supplementary Material; Table S1). The *time since intervention* coefficient showed a significant post-failure slope change only at monitoring stations RD019, RD023, RD033, and RD035, indicating that their turbidity records showed monthly decreases of ~ 1.79%–2.0% (Supplementary Material; Table S1). For the remaining monitoring stations, the *time since intervention* coefficient was not significant (Supplementary Material; Table S1); therefore, we could not reject the null hypothesis of no post-failure slope change relative to the pre-failure slope change.

**Fig. 4:**
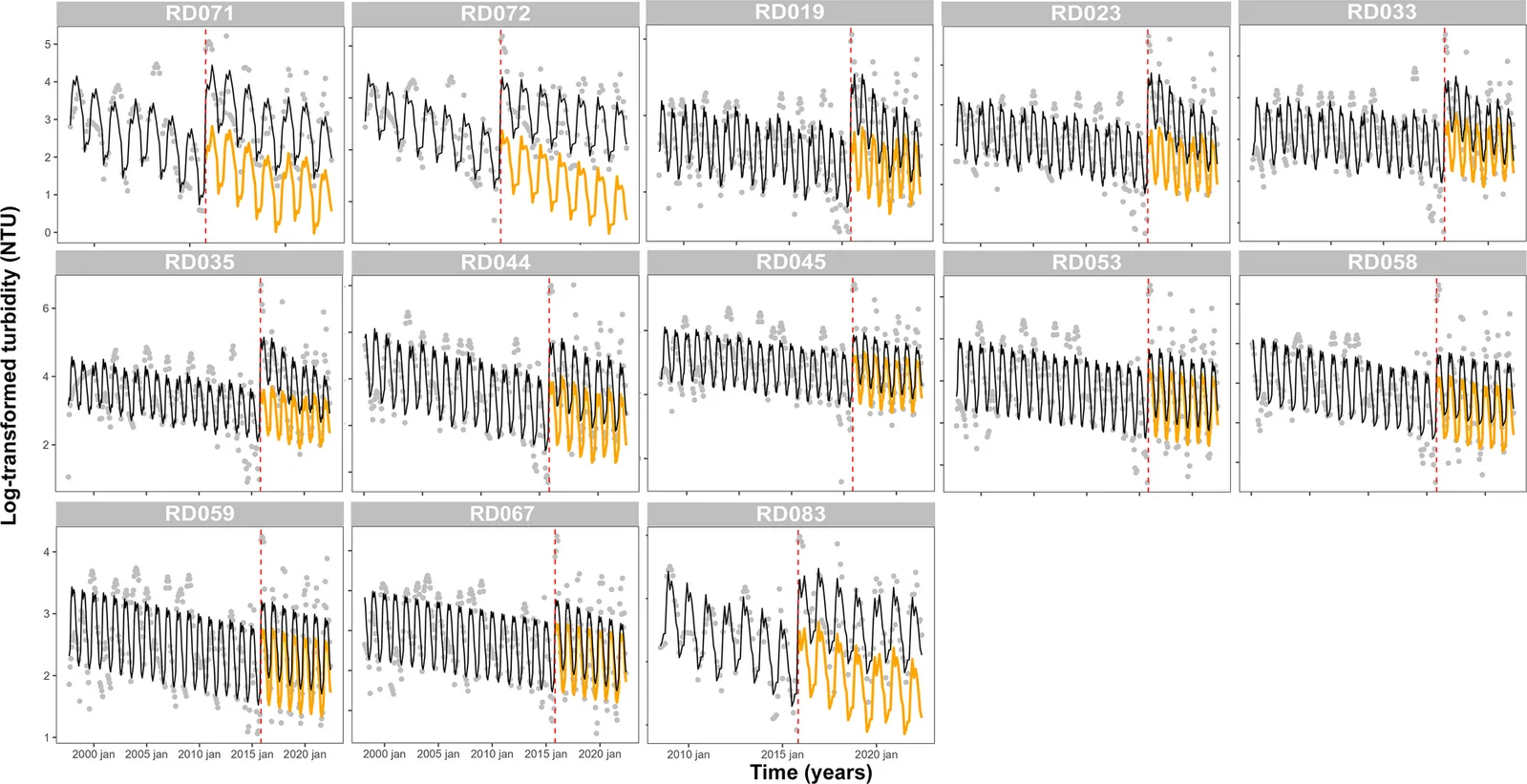
Observed and modeled monthly turbidity at each monitoring station along the Doce River. grey points show the original monthly observations. Solid black lines show the fitted interrupted time-series (ITS) model including the Fundão dam failure. orange lines show the corresponding counterfactual “no-impact” trajectories predicted from the pre-failure dynamics. the red dashed line marks the year of the collapse (2015)

The counterfactual-based models, which are the same ITS model with the intervention coefficient slope set to zero, indicated that the post-failure time series diverged from the turbidity values predicted in the absence of the event (Fig. 4). Nevertheless, visual inspection of the counterfactual trajectories (Fig. 4) suggested that, at the most downstream stations (i.e., RD045–RD067), the observed turbidity in the most recent years approached levels expected under natural dynamics.

Across stations, Cohen’s *d* ranged from 0.64 (RD067) to 2.75 (RD072) (Table 1). Effect sizes were large (*d* > 0.8) at 11 of the 13 monitoring stations and moderate (0.5 < *d* < 0.8) at RD053 and RD067 (Table 1). All effect size estimates had 95% confidence intervals above zero and were statistically significant (p ≤ 0.003; Table 1). The spatial pattern of effect sizes was consistent with the attenuation of turbidity along the river continuum. Stations located closest to the Fundão dam exhibited the strongest responses, whereas more distal stations showed progressively smaller, though still mostly large effects (e.g., *d* ≈ 0.85–1.71 at RD019–RD059). The two most downstream stations, RD053 and RD067, had only moderate effect sizes (*d* = 0.64–0.69).

**Table 1:** Effect size (Cohen’s *d*) for the impact of the Fundão tailings dam failure on turbidity at each monitoring station along the Doce River. distances (km) were measured downstream from the dam. shown are point estimates of Cohen’s *d*, their 95% confidence intervals (2.5%–97.5%) and p-values from the interrupted time-series counterfactual model

Distance (km)	Station	Cohen’s *d*	2.5% CI	97.5% CI	p-value
64.90	RD071	2.070	1.308	2.681	< 0.001
85.50	RD072	2.745	1.970	3.286	< 0.001
143.30	RD019	1.499	1.044	1.910	< 0.001
197.70	RD023	1.710	1.263	2.105	< 0.001
240.10	RD035	1.834	1.321	2.306	< 0.001
269.10	RD033	1.242	0.786	1.657	< 0.001
310.90	RD083	2.027	1.370	2.581	< 0.001
346.30	RD044	1.348	0.847	1.766	< 0.001
361.00	RD045	0.850	0.395	1.287	< 0.001
387.70	RD053	0.687	0.224	1.126	0.003
421.20	RD058	1.010	0.489	1.464	< 0.001
454.80	RD059	0.862	0.418	1.280	< 0.001
490.10	RD067	0.635	0.203	1.024	0.003

## Discussion

In this study, we found that turbidity recovery in the Doce River during the 7 years following the Fundão tailings dam failure was partial and constrained in both time and space along the mainstem. Seasonal-Trend Loess (STL), Interrupted Time Series (ITS) models, and Cohen’s *d* effect sizes all pointed to the same overarching pattern: the impact of the Fundão dam failure on turbidity was strong and persisted throughout the 2015–2022 post-failure period. STL decomposition showed an abrupt post-failure increase in seasonal amplitude, with sharper dry–wet contrasts immediately after collapse and only gradual attenuation over time, without a complete return to the pre-failure seasonal pattern. The combination of ITS counterfactual trajectories and Cohen’s *d* effect sizes consistently indicated a longitudinal attenuation of turbidity along the river continuum over time. Overall, our findings suggest that the evaluated reaches of the Doce River were recovering, but full recovery of the turbidity conditions had not yet occurred by 2022.

Previous studies have suggested that many physicochemical parameters of Doce River water, including turbidity, had returned to pre-failure levels before 2022 (da Cunha Richard et al., 2020; Garcia et al., 2024; Silva et al., 2022; Wild et al., 2024). Using a more conservative framework based on highly recommended seasonal decomposition (Huang et al., 2017; Schäfer et al., 2021), interrupted time-series models (Wauchope et al., 2021) and their counterfactual trajectories (Grace et al., 2021; O’Garra et al., 2025), we showed that by 2022, turbidity recovery remained partial, seasonal, and spatially heterogeneous in the Doce River. Here, we define Doce River recovery as the complete or near-complete disappearance of the impact signal when the entire post-failure time series is compared with an explicit counterfactual trajectory that accounts for both the long-term trend and seasonal structure. Those previous studies, however, understand it as the turbidity post-failure values lying within or close to the historical, pre-failure background levels. Given these divergent, method-dependent conclusions, we recommend adopting a precautionary approach when evaluating post-failure recovery. This implies that optimistic assessments of full recovery should be considered with caution, and long-term monitoring and management actions should be maintained until deviations from pre-failure dynamics become negligible—especially, during the wet season.

In mine tailing-impacted rivers, a marked seasonal pattern in turbidity, with higher values during the wet season, is widespread (Fonseca et al., 2023; Rutherfurd et al., 2020; Wild et al., 2024). Accordingly, we verified that turbidity remained much higher during the rainy season across all monitoring stations, indicating that the residual impact was concentrated in wet months. During the study period, the magnitude and dynamics of the turbidity contrast between wet-season peaks and dry-season troughs were highly station-specific, with no clear pattern in relation to the distance from the dam failure. However, we verified that these post-failure seasonal contrasts did not return to their pre-failure conditions at any station. Observing the extent to which turbidity deviates from pre-failure levels in the dry and wet seasons may be an important proxy for assessing Doce River recovery. These persistent, station-specific seasonal turbidity anomalies were also reflected in ITS-based counterfactual trajectories and effect sizes.

The ITS models and their counterfactual trajectories showed that, although post-failure turbidity remained above expected levels at most stations, convergence towards the counterfactual was most evident at downstream sites, where Cohen’s *d* effect sizes were moderate. In other words, the most downstream stations in the evaluated reach were likely closer to pre-failure turbidity conditions, but residual anomalies in wet-season values were still sufficient to produce detectable departures from the counterfactual over the entire post-failure period. At first, the evidence that most downstream stations may be closer to pre-failure turbidity seems to diverge from the results obtained from the ITS slopes, which showed that ongoing recovery occurred only at stations RD019, RD023, RD033, and RD035—which did not fall within this further downstream reach. However, the ITS slope results indicate that a substantial reduction in turbidity was statistically detected only at stations RD019, RD023, RD033, and RD035, which does not necessarily imply that it did not recover at the remaining stations; it only means that its variation was either too weak or too noisy to be detected by our ITS model during the study period. We argue that a more pronounced turbidity recovery at the most downstream monitoring stations can be expected because most of the tailing mass was deposited within the first 120 km downstream (Carmo et al., 2017). The very high turbidity in the upper Doce River decreased progressively with tributary dilution further downstream (Hatje et al., 2017).

Despite the growing recognition of the importance of site-specific seasonal baselines, interrupted time-series analyses, and comparisons of observed trajectories with counterfactuals when evaluating environmental impacts, to our knowledge, this study is the first to apply this integrated framework to quantify turbidity impacts and recovery after one of the most severe environmental disasters in global mining, the Fundão tailing dam failure. However, our study has some limitations. Our data analysis was constrained to a single, albeit the most conservative, physicochemical variable available in the IGAM historical dataset, and we are aware that recovery time is variable-dependent (Silva et al., 2022; Wild et al., 2024). In addition, because IGAM data come from an observational monitoring network rather than a purpose-designed impact experiment, some confounding influences cannot be entirely ruled out from our results. We could not reliably estimate the time required for turbidity to return to pre-failure levels because the ITS model did not consistently capture a significant post-failure decline at most stations, likely reflecting the intrinsic limitations of the method and model (i.e., a short post-impact period and high temporal variability). Nevertheless, our results can support concrete recommendations for the long-term monitoring and management of the Doce River.

Turbidity recovery is partial and heterogeneous in time and space; therefore, post-failure evaluation in the Doce River—and likely other tailing-impacted rivers— cannot rely solely on short-term snapshots or dry-season measurements. The persistence of elevated wet-season turbidity and strong spatial heterogeneity along the mainstem indicates that monitoring programs should maintain high-frequency, long-term measurements, with particular emphasis on the rainy months and stations closest to the Fundão Dam. We suggest that seasonal deviations in turbidity from pre-failure levels may provide a valuable proxy for assessing the recovery of the Doce River.

## Conclusions

Over the seven years following the Fundão tailing dam failure, turbidity in the Doce River showed evidence of recovery; however, this recovery was only partial and strongly heterogeneous in time and along the main river stem. The impact of the disaster remained pronounced throughout the study period, with dry–wet contrasts in turbidity much stronger than before the dam failure and only gradually attenuating. Overall, the evaluated reaches of the Doce River did not return to pre-failure turbidity conditions by 2022.

## Supplementary Information

Below is the link to the electronic supplementary material.

Supplementary file1 (DOCX 1371 kb)

## Data Availability

Turbidity data were obtained from the IGAM InfoHidro platform (http://portalinfohidro.igam.mg.gov.br).
