# Supplementary material for: A seasonally explicit counterfactual framework reveals incomplete turbidity recovery in a tropical river seven years after a major mine-tailings disaster
Source: Environ Monit Assess. 2026 Aug 20;198(9):968. doi: 10.1007/s10661-026-15706-2 (PMC13493438; doi:10.1007/s10661-026-15706-2)
Supplement: Supplementary file 1 [file 10661_2026_15706_MOESM1_ESM.docx]

**Supplementary material**

**Figure S1.** Annual mean turbidity for the monitoring stations located along the path of the mine tailings plume in the Doce River (southeastern Brazil). The monitoring stations are ordered from the closest to the Fundão dam failure site (RD011) to the farthest downstream station (RD067). The red dashed line indicates the year of dam collapse (2015).

**Figure S2.** Annual mean turbidity in the dry (left panel) and rainy (right panel) seasons for monitoring stations located in the Doce River reach impacted by the Fundão Dam collapse. The red dashed line indicates the year of dam failure (2015).

**Table S1.** Interrupted Time Series (ITS) models for each monitoring station. Only statistically significant Fourier seasonal terms were retained in the model summaries (S1.12, S2.12, S3.12, S4.12, C1.12, C2.12, and C4.12). All coefficients are on a logarithmic scale. To obtain the percent change in *Y* per unit change in *X*, we applied (exp(β) -1) × 100. To quantify the multiplicative (fold) change in *Y* per unit change in *X*, we applied exp(β). The monitoring stations are ordered from the closest to the Fundão dam failure site (RD071) to the farthest downstream station (RD067).

| **Station** | **Term** | **Estimate** | **Std. Error** | **t-value** | **p-value** | **R^2^** | **F-statistic** | **p-value** |
| --- | --- | --- | --- | --- | --- | --- | --- | --- |
| RD071 | Intercept | 4.28 | 0.25 | 16.90 | <0.001 | 0.56 | 13.48 | <0.001 |
|  | Time | -0.02 | 0.01 | -4.56 | <0.001 |  |  |  |
|  | Intervention | 2.82 | 0.36 | 7.83 | <0.001 |  |  |  |
|  | Time since Intervention | -0.01 | 0.01 | -1.16 | 0.250 |  |  |  |
|  | S1.12 | 0.65 | 0.13 | 5.15 | <0.001 |  |  |  |
|  | C1.12 | -1.14 | 0.13 | -8.96 | <0.001 |  |  |  |
| RD072 | Intercept | 4.78 | 0.27 | 17.49 | <0.001 | 0.57 | 14.31 | <0.001 |
|  | Time | -0.03 | 0.01 | -5.98 | <0.001 |  |  |  |
|  | Intervention | 2.96 | 0.39 | 7.62 | <0.001 |  |  |  |
|  | Time since Intervention | 0.01 | 0.01 | 1.20 | 0.230 |  |  |  |
|  | S1.12 | 0.87 | 0.14 | 6.33 | <0.001 |  |  |  |
|  | C1.12 | -1.17 | 0.14 | -8.47 | <0.001 |  |  |  |
|  | S3.12 | -0.31 | 0.14 | -2.29 | 0.020 |  |  |  |

| **Station** | **Term** | **Estimate** | **Std. Error** | **t-value** | **p-value** | **R^2^** | **F-statistic** | **p-value** |
| --- | --- | --- | --- | --- | --- | --- | --- | --- |
| RD019 | Intercept | 4.14 | 0.13 | 31.55 | <0.001 | 0.51 | 21.05 | <0.001 |
|  | Time | -0.01 | 0.00 | -4.62 | <0.001 |  |  |  |
|  | Intervention | 2.28 | 0.25 | 9.06 | <0.001 |  |  |  |
|  | Time since Intervention | -0.02 | 0.01 | -4.40 | <0.001 |  |  |  |
|  | S1.12 | 0.20 | 0.08 | 2.55 | 0.010 |  |  |  |
|  | C1.12 | -1.07 | 0.08 | -13.45 | <0.001 |  |  |  |
| RD023 | Intercept | 4.30 | 0.12 | 36.42 | <0.001 | 0.56 | 25.66 | <0.001 |
|  | Time | -0.01 | 0.00 | -5.51 | <0.001 |  |  |  |
|  | Intervention | 2.40 | 0.23 | 10.61 | <0.001 |  |  |  |
|  | Time since Intervention | -0.02 | 0.00 | -5.01 | <0.001 |  |  |  |
|  | S1.12 | 0.16 | 0.07 | 2.24 | 0.030 |  |  |  |
|  | C1.12 | -1.02 | 0.07 | -14.30 | <0.001 |  |  |  |
|  | S4.12 | 0.18 | 0.07 | 2.59 | 0.010 |  |  |  |
| RD035 | Intercept | 4.51 | 0.12 | 37.79 | <0.001 | 0.50 | 20.35 | <0.001 |
|  | Time | -0.01 | 0.00 | -5.59 | <0.001 |  |  |  |
|  | Intervention | 2.22 | 0.23 | 9.71 | <0.001 |  |  |  |
|  | Time since Intervention | -0.02 | 0.00 | -4.40 | <0.001 |  |  |  |
|  | S1.12 | -0.37 | 0.07 | -5.12 | <0.001 |  |  |  |
|  | C1.12 | -0.82 | 0.07 | -11.35 | <0.001 |  |  |  |
|  | S2.12 | -0.18 | 0.07 | -2.47 | 0.010 |  |  |  |

| **Station** | **Term** | **Estimate** | | **Std. Error** | | **t-value** | | **p-value** | | **R^2^** | | **F-statistic** | | **p-value** | |
| --- | --- | --- | --- | --- | --- | --- | --- | --- | --- | --- | --- | --- | --- | --- | --- |
| RD033 | Intercept | 4.17 | | 0.12 | | 33.48 | | <0.001 | | 0.50 | | 20.61 | | <0.001 | |
|  | Time | 0.00 | | 0.00 | | -3.06 | | <0.001 | |  |  |  |  |  |  |
|  | Intervention | 1.80 | | 0.24 | | 7.53 | | <0.001 | |  |  |  |  |  |  |
|  | Time since Intervention | -0.02 | | 0.01 | | -3.96 | | <0.001 | |  |  |  |  |  |  |
|  | S1.12 | -0.69 | | 0.08 | | -9.20 | | <0.001 | |  |  |  |  |  |  |
|  | C1.12 | -0.82 | | 0.08 | | -10.90 | | <0.001 | |  |  |  |  |  |  |
| RD083 | Intercept | 4.23 | | 0.19 | | 21.89 | | <0.001 | | 0.60 | | 16.76 | | <0.001 | |
|  | Time | -0.02 | | 0.00 | | -4.85 | | <0.001 | |  |  |  |  |  |  |
|  | Intervention | 2.08 | | 0.28 | | 7.48 | | <0.001 | |  |  |  |  |  |  |
|  | Time since Intervention | 0.00 | | 0.01 | | 0.02 | | 0.990 | |  |  |  |  |  |  |
|  | S1.12 | -0.86 | | 0.10 | | -8.75 | | <0.001 | |  |  |  |  |  |  |
|  | C1.12 | -0.84 | | 0.10 | | -8.54 | | <0.001 | |  |  |  |  |  |  |
|  | C4.12 | 0.23 | | 0.10 | | 2.38 | | 0.020 | |  |  |  |  |  |  |
| RD044 | Intercept | 4.45 | | 0.14 | | 31.05 | | <0.001 | | 0.51 | | 19.19 | | <0.001 | |
|  | Time | -0.01 | | 0.00 | | -6.51 | | <0.001 | |  |  |  |  |  |  |
|  | Intervention | 1.57 | | 0.26 | | 6.02 | | <0.001 | |  |  |  |  |  |  |
|  | Time since Intervention | -0.01 | | 0.01 | | -1.44 | | 0.150 | |  |  |  |  |  |  |
|  | S1.12 | 0.36 | | 0.08 | | 4.26 | | <0.001 | |  |  |  |  |  |  |
|  | C1.12 | 1.12 | | 0.08 | | 13.19 | | <0.001 | |  |  |  |  |  |  |
|  | S2.12 | -0.18 | | 0.08 | | -2.11 | | 0.040 | |  |  |  |  |  |  |
|  | C4.12 | -0.23 | | 0.08 | | -2.74 | | 0.010 | |  |  |  |  |  |  |
| **Station** | **Term** | **Estimate** | **Std. Error** | | **t-value** | | **p-value** | | **R^2^** | | **F-statistic** | | **p-value** | |  |
| RD045 | Intercept | 4.24 | 0.14 | | 29.84 | | <0.001 | | 0.46 | | 17.68 | | <0.001 | |  |
|  | Time | -0.01 | 0.00 | | -4.59 | | <0.001 | |  |  |  |  |  |  |  |
|  | Intervention | 1.11 | 0.27 | | 4.08 | | <0.001 | |  |  |  |  |  |  |  |
|  | Time since Intervention | -0.01 | 0.01 | | -1.47 | | 0.140 | |  |  |  |  |  |  |  |
|  | S1.12 | -0.89 | 0.09 | | -10.39 | | <0.001 | |  |  |  |  |  |  |  |
|  | C1.12 | -0.84 | 0.09 | | -9.81 | | <0.001 | |  |  |  |  |  |  |  |
| RD053 | Intercept | 4.17 | 0.15 | | 28.23 | | <0.001 | | 0.45 | | 16.60 | | <0.001 | |  |
|  | Time | 0.00 | 0.00 | | -4.04 | | <0.001 | |  |  |  |  |  |  |  |
|  | Intervention | 0.92 | 0.28 | | 3.25 | | 0.001 | |  |  |  |  |  |  |  |
|  | Time since Intervention | -0.01 | 0.01 | | -0.94 | | 0.350 | |  |  |  |  |  |  |  |
|  | S1.12 | 0.30 | 0.09 | | 3.40 | | <0.001 | |  |  |  |  |  |  |  |
|  | C1.12 | -1.21 | 0.09 | | -13.55 | | <0.001 | |  |  |  |  |  |  |  |
|  | C2.12 | -0.19 | 0.09 | | -2.16 | | 0.030 | |  |  |  |  |  |  |  |
| RD058 | Intercept | 4.42 | 0.16 | | 28.06 | | <0.001 | | 0.49 | | 17.62 | | <0.001 | |  |
|  | Time | -0.01 | 0.00 | | -5.35 | | <0.001 | |  |  |  |  |  |  |  |
|  | Intervention | 1.15 | 0.29 | | 4.01 | | <0.001 | |  |  |  |  |  |  |  |
|  | Time since Intervention | 0.00 | 0.01 | | -0.35 | | 0.730 | |  |  |  |  |  |  |  |
|  | S1.12 | 0.45 | 0.09 | | 4.87 | | <0.001 | |  |  |  |  |  |  |  |
|  | C1.12 | 1.22 | 0.09 | | 13.13 | | <0.001 | |  |  |  |  |  |  |  |
|  | C4.12 | -0.25 | 0.09 | | -2.64 | | 0.010 | |  |  |  |  |  |  |  |

| **Station** | **Term** | **Estimate** | **Std. Error** | **t-value** | **p-value** | **R^2^** | **F-statistic** | **p-value** |
| --- | --- | --- | --- | --- | --- | --- | --- | --- |
| RD059 | Intercept | 4.32 | 0.15 | 28.45 | <0.001 | 0.47 | 17.68 | <0.001 |
|  | Time | -0.01 | 0.00 | -4.98 | <0.001 |  |  |  |
|  | Intervention | 1.22 | 0.29 | 4.20 | <0.001 |  |  |  |
|  | Time since Intervention | -0.01 | 0.01 | -1.32 | 0.190 |  |  |  |
|  | S1.12 | 0.23 | 0.09 | 2.52 | 0.010 |  |  |  |
|  | C1.12 | -1.28 | 0.09 | -13.92 | <0.001 |  |  |  |
|  | S4.12 | 0.19 | 0.09 | 2.07 | 0.040 |  |  |  |
| RD067 | Intercept | 4.22 | 0.15 | 28.43 | <0.001 | 0.51 | 20.56 | <0.001 |
|  | Time | -0.01 | 0.00 | -5.01 | <0.001 |  |  |  |
|  | Intervention | 1.12 | 0.28 | 3.99 | <0.001 |  |  |  |
|  | Time since Intervention | -0.01 | 0.01 | -1.75 | 0.080 |  |  |  |
|  | S1.12 | -0.28 | 0.09 | -3.10 | <0.01 |  |  |  |
|  | C1.12 | 1.34 | 0.09 | 15.05 | < 2e-16 |  |  |  |

**Table S2.** Augmented Dickey–Fuller (ADF) test for stationarity of monthly turbidity time series at each monitoring station in the Doce River.

| **Station** | **τ (ADF)** | **1% critical value** | **p-value** | **Stationarity** |
| --- | --- | --- | --- | --- |
| RD019 | -7.52 | -2.58 | <0.001 | Yes |
| RD023 | -7.67 | -2.58 | <0.001 | Yes |
| RD033 | -8.38 | -2.58 | <0.001 | Yes |
| RD035 | -8.45 | -2.58 | <0.001 | Yes |
| RD044 | -6.65 | -2.58 | <0.001 | Yes |
| RD045 | -7.81 | -2.58 | <0.001 | Yes |
| RD053 | -7.25 | -2.58 | <0.001 | Yes |
| RD058 | -8.15 | -2.58 | <0.001 | Yes |
| RD059 | -8.56 | -2.58 | <0.001 | Yes |
| RD067 | -8.80 | -2.58 | <0.001 | Yes |
| RD071 | -5.41 | -2.58 | <0.001 | Yes |
| RD072 | -6.95 | -2.58 | <0.001 | Yes |
| RD083 | -5.60 | -2.58 | <0.001 | Yes |
